# An Overview of Long-Acting GnRH Agonists in Premenopausal Breast Cancer Patients: Survivorship Challenges and Management

**DOI:** 10.3390/curroncol31080314

**Published:** 2024-07-25

**Authors:** Nathalie LeVasseur, Mita Manna, Katarzyna J. Jerzak

**Affiliations:** 1 BC Cancer Vancouver Centre, University of British Columbia, Vancouver, BC V5Z 4E6, Canada; 2 Department of Medicine and Department of Oncology, University of Saskatchewan, Saskatoon, SK S7N 5E5, Canada; mita.manna@saskcancer.ca; 3 Sunnybrook Odette Cancer Centre, University of Toronto, Toronto, ON M4N 3M5, Canada; katarzyna.jerzak@sunnybrook.ca

**Keywords:** goserelin, GnRH, LHRH agonist, breast cancer adjuvant therapy, ovarian function suppression, hormone receptor-positive breast cancer, premenopausal, long-acting, survivorship

## Abstract

Managing breast cancer in premenopausal women poses unique challenges due to its considerable effect on both morbidity and mortality. Goserelin, a gonadotropin-releasing hormone agonist, has emerged among the various modalities as a preferred option for ovarian function suppression, owing to its efficacy in reducing ovarian estrogen production in premenopausal women with hormone receptor-positive breast cancer. Recent studies have affirmed the efficacy and safety of long-acting (LA) goserelin 10.8 mg every 12 weeks, offering comparable outcomes to monthly injections. This flexibility enables personalized treatment approaches, potentially enhancing patient satisfaction. Off-label utilization of goserelin LA surged during the coronavirus disease pandemic, prompting initiatives to broaden its use for breast cancer treatment. Switching to goserelin LA can streamline treatment, boost adherence, and optimize resource utilization. With the recent approval of goserelin 10.8 mg LA by Health Canada on 6 May 2024, for use in breast cancer, Canada is the latest to join over 60 countries worldwide to expand the accepted indications for goserelin LA and ensure its availability to potentially enhance healthcare delivery, patient care, and breast cancer outcomes. Goserelin LA offers premenopausal patients a means to more effectively manage the constraints imposed by breast cancer treatment and its impact on survivorship.

## 1. Introduction

### 1.1. Epidemiology

Breast cancer remains one of the leading cancer diagnoses globally and impacts over 29,000 Canadian women every year [1,2]. Although the majority of breast cancer cases affect women aged 50 and above, approximately one-fourth of cases occur in younger, premenopausal women [1]. The hormone receptor (HR)-positive and human epidermal growth factor receptor 2 (HER2)-negative breast cancer subtype represents 60–70% of incident cases [3]. In HR-positive breast cancer, it is primarily the activation of estrogen receptors (ER) that drives tumor growth and progression by promoting gene transcription, leading to abnormal cell proliferation [4]. Treatment commonly targets inhibition of estrogen/ER signaling through anti-estrogen or endocrine therapy (ET) [4].

### 1.2. Current Management of Early-Stage HR-Positive Breast Cancer

For pre-menopausal patients with lymph node-negative, HR-positive breast cancer, the use of genomic assays may be used to predict potential benefits from chemotherapy and provide prognostic information [5]. Among patients with lymph node-positive HR-positive disease, A Clinical Trial RX for Positive Node, Endocrine Responsive Breast Cancer (RxPONDER) [6] and Microarray in Node-Negative and 1 to 3 Positive Lymph Node Disease May Avoid Chemotherapy [7,8] trials demonstrated a benefit of ~5% absolute reduction in relapse in pre-menopausal women, irrespective of the genomic score result, suggesting broad benefit from chemotherapy in the pre-menopausal patient population. However, whether the benefit is due to its cytotoxic effects versus the induction of menopause in pre-menopausal women is unclear. The ongoing Ovarian Function Suppression plus Endocrine Therapy (OFSET) study is being conducted to answer this question [9].

**Figure 1:**
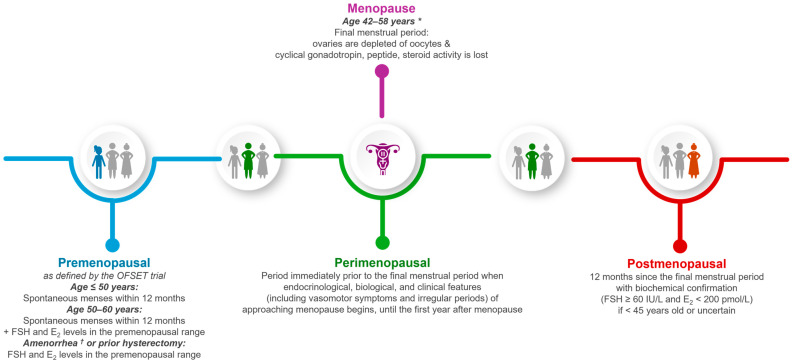
Definitions of pre-, peri-, and post-menopausal [10,11,15,24,25,26]. E_2_, estradiol; FSH, follicle stimulating hormone; OFSET, Ovarian Function Suppression plus Endocrine Therapy. * Varies based on health and socioeconomic factors (e.g., oral contraceptive, alcohol, smoking, diet, physical activity, weight, ethnicity, education, employment, environmental exposure). ^†^ Due to an intrauterine device or prior uterine ablation.

Current ET recommendations for premenopausal and postmenopausal breast cancer patients (see definitions in Figure 1) differ primarily in the choice of adjuvant ET [10,11,12,13]. Factors that impact selection include clinicopathological features, disease biology, comorbidities, family planning wishes, mitigation of long-term treatment complications, and patient preference [10,11,12,13]. ET for premenopausal breast cancer patients typically includes tamoxifen alone or a gonadotropin-releasing hormone (GnRH) agonist as ovarian function suppression (OFS) therapy in combination with ET, depending on various factors as outlined above [11,14,15,16,17,18,19]. The combination of aromatase inhibitor (AI) + OFS is favored for best disease-free survival (DFS) benefits. However, complete OFS with profound suppression of hormone levels is required for the antitumor effects since AI alone can stimulate ovarian function [20,21]. Studies, including the Suppression of Ovarian Function Trial (SOFT) and the Tamoxifen and Exemestane Trial (TEXT), have supported the addition of OFS to ET in pre-menopausal patients, as reflected in the NCCN Clinical Practice Guidelines in Oncology (NCCN Guidelines^®^) for Breast Cancer, American Society of Clinical Oncology guidelines, and European Society for Medical Oncology guidelines [11,14,15,16,17,18,19,21]. A meta-analysis of 25 randomized trials involving nearly 15,000 premenopausal women with ER-positive or ER-unknown breast cancer showed that ovarian ablation or OFS significantly reduced the 15-year risk of recurrence (rate ratio [RR] = 0.63 for women <45 years old) and mortality (RR = 0.69) [22]. When a patient wants to attempt pregnancy, ET can temporarily be interrupted without fear of increasing the short-term 3-year risk of breast cancer events or distant relapse-free interval (DRFI), according to the Pregnancy Outcome and Safety of Interrupting Therapy for Women with Endocrine Responsive Breast Cancer trial [23].

The aim of this perspective article is to contextualize the survivorship challenges faced by premenopausal women with breast cancer who undergo ovarian function suppression. Here, we emphasize how the current monthly regimen imposes a significant burden on both the healthcare system and the patients. Our objectives are to review the current literature and data and to illustrate scenarios where long-acting formulations of OFS can directly address survivorship issues using real-world cases.

### 1.3. Ongoing Challenges in Premenopausal Breast Cancer Management

#### 1.3.1. Side Effects of OFS

Younger breast cancer patients tend to be diagnosed with more aggressive tumors, with higher rates of recurrence and inferior outcomes compared to older individuals [27]. Despite advancements in therapy, significant challenges persist, particularly with the integration of OFS into adjuvant ET for premenopausal women [28,29]. Side effects of OFS may contribute to patients being less adherent to treatment over time, especially among younger patients with favorable performance status [28,29]. In the SOFT trial, adverse events of grade 3 or higher were more common in groups receiving OFS: 24.6% for tamoxifen vs. 31.0% for tamoxifen + OFS and 32.3% for an AI (exemestane) + OFS [30]. Hot flushes were more frequently reported in groups receiving OFS compared to those on tamoxifen alone (7.8% vs. 12.2% vs. 10.1%, respectively) [30]. AI + OFS also had the highest incidence of musculoskeletal symptoms (11.4% vs. 6.7% for tamoxifen vs. 5.7% for tamoxifen + OFS) [30].

#### 1.3.2. Survivorship Challenges

**Figure 2:**
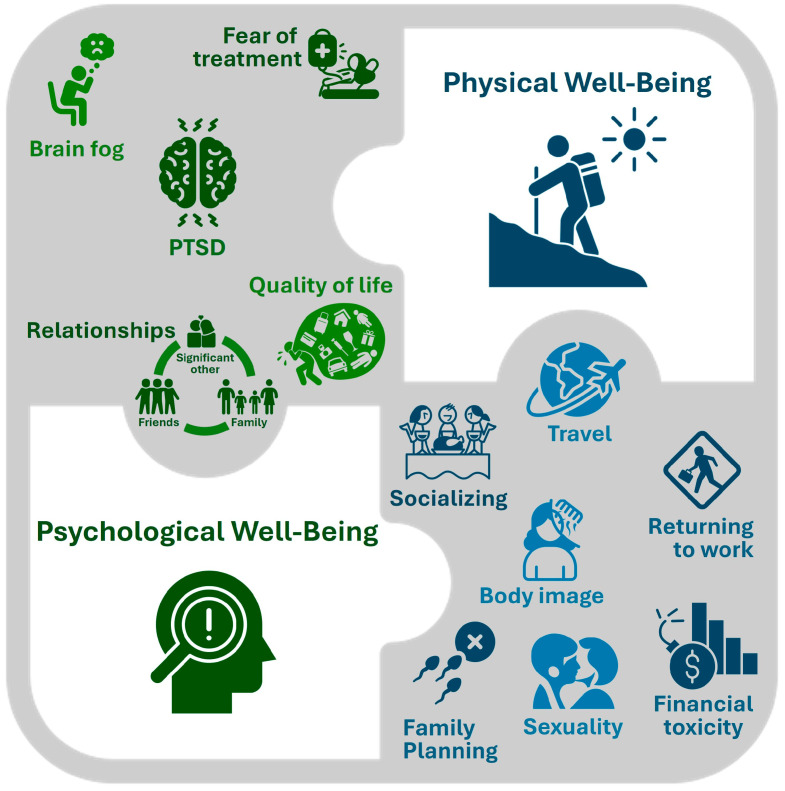
Survivorship challenges faced by premenopausal women with breast cancer [12,31,32,33,34,35,36,37,38]. PTSD, posttraumatic stress disorder. Adapted from TheNounProject.com icons using Creative Commons Attribution License (CC BY 3.0).

Breast cancer patients encounter unique and persistent survivorship challenges impacting their physical, mental, and psychosocial well-being, including decreased energy levels, brain fog, mood changes, body image, posttraumatic stress from frequent clinic/treatment visits, financial toxicity, challenges in returning to work/school, sexuality, family planning, and early parenthood, amongst others (Figure 2) [12,31,32,33,34,35,36,37]. Women with early-stage breast cancer may be more vulnerable to the physical and emotional toll of undergoing treatment with a long horizon ahead of them and have expressed concerns about their quality of life, independence, and note that relationships with family members/partners/friends and daily activities may be affected [38].

Patients undergoing cancer treatment generally prioritize spending time with loved ones, reducing wait times for treatment, opting for convenient subcutaneous (SC) administration, and engaging in shared decision-making with physicians [38]. They express concerns about how treatment may affect their ability to enjoy hobbies, attend events, socialize, fulfill family obligations, be with loved ones, and travel, which are crucial for maintaining normalcy and emotional well-being [38]. RUBY, or Reducing the Burden of Breast Cancer in Young Women, is a pan-Canadian research study that examines breast cancer in young women (40 years and under) in Canada [27]. In doing so, the unique challenges that women in this age group face can be identified, and best practices for their medical care can be determined.

## 2. Management of Ovarian Function Suppression with GnRH Agonists Administered Every 3 Months in Breast Cancer

Ovarian function suppression can be achieved via three different modalities: ovarian irradiation, surgical oophorectomy, or medical suppression [39]. Ovarian irradiation was commonly used prior to 1980 but has since fallen out of favor [39]. Bilateral salpingo-oophorectomy (BSO) is a minimally invasive yet irreversible procedure [39]. Reversible medical suppression with long-acting GnRH agonist therapy may be more appealing to young women [39].

Long-acting GnRH agonist formulations available in Canadian formularies include goserelin and leuprolide. Studies included in this review were identified primarily from a PubMed search targeting GnRH agonist (goserelin OR leuprolide/leuprorelin) AND breast cancer AND long-acting (3-month OR 12-weeks OR 6-month OR 24-weeks) AND efficacy and screened for relevancy, supplemented with additional unpublished study data available in the public domain. The focus of this paper is primarily on goserelin LA 10.8 mg in support of its recent approval by Health Canada on 6 May 2024 [40]. Data supporting the use of long-acting leuprolide 11.25 mg and 22.5 mg are summarized in Supplementary Tables S1 and S2 [41,42,43,44,45,46,47,48,49,50]. Triptorelin data was excluded, as it has been removed from most Canadian formularies.

### 2.1. Goserelin Dosage Options and Guidelines for Use

Goserelin functions as a GnRH agonist, effectively reducing ovarian estrogen production, the primary estrogen source in premenopausal women [51]. Approved in the United States and Canada in 1989, this medication is available as a SC slow-release implant with two dosage options: 3.6 mg every 28 days or 10.8 mg every 12 weeks, both with approved indications for prostate cancer, breast cancer, and endometriosis [40,52,53,54].

The NCCN Guidelines^®^ for Breast Cancer support both formulations of goserelin, 3.6 mg every 4 weeks and 10.8 mg every 12 weeks, for OFS in premenopausal patients with HR-positive breast cancer [11]. Combining goserelin 3.6 mg with tamoxifen has shown superior efficacy to goserelin 3.6 mg alone, and at least equivalent efficacy to cyclophosphamide, methotrexate, and 5-fluorouracil (CMF) chemotherapy [55].

OFS initiation can be considered with neoadjuvant or adjuvant chemotherapy for the purpose of fertility preservation. However, preservation of ovarian function is not guaranteed as it only slightly improves the chances of fertility (10.3% for GnRH agonists versus 5.5% for control, *p* = 0.03) [11,56].

In cases where chemotherapy is not planned, the NCCN Guidelines for Breast Cancer indicate OFS alone or combined with tamoxifen should be initiated for 1 to 2 cycles until estradiol (E_2_) levels reach postmenopausal levels, at which time an AI could be considered [11]. In clinical practice, a postmenopausal state is not always achieved with OFS since a proportion of patients have persistently elevated E_2_ levels despite achieving amenorrhea [15]. The ASCO guidelines on ovarian suppression do not recommend routine monitoring of E_2_ levels but rather monitoring for physiological changes (e.g., resumption of menses, cyclical fluctuations of climacteric symptoms) that may suggest ovarian function recovery [15].

Ovarian escape has been reported more often among women who were younger rather than older or overweight rather than normal weight based on a retrospective, real-world study, a case series, and an estrogen substudy of SOFT [57,58,59]. The latter is not surprising since adipose tissue serves as the primary source of serum estrogen, and overweight patients tend to have larger reserves of estrogen precursors [60]. Higher risks of ovarian escape with OFS may be related to the influence of E_2_ levels on host factors among younger women that render the disease biology slightly different than in older women with HR-positive breast cancer [57].

### 2.2. Efficacy Studies of Goserelin 10.8 mg LA in Breast Cancer

Multiple studies indicate that goserelin 10.8 mg LA every 3 months is just as effective as goserelin 3.6 mg every month in achieving and maintaining OFS in premenopausal women with breast cancer (Table 1 and Table 2) [50,51,55,61,62]. There is evidence to suggest that SC depot injections are more comfortable and convenient for patients if they can be transitioned from a monthly to an every-3-month dosing schedule with goserelin 10.8 mg LA, potentially improving adherence [51,55,61]. With longer intervals between doses, this regimen could diminish the need for frequent clinic visits, thus alleviating resource utilization and reducing healthcare spending [51].

In a phase II study conducted in Japan, Masuda et al. compared the efficacy and safety of goserelin 10.8 mg LA every 3 months versus goserelin 3.6 mg every month in premenopausal women with ER-positive early breast cancer (Table 1) [55]. The primary endpoint of the geometric mean E_2_ area under the serum concentration-time curve from week 4 to week 24 [AUC_(4–24 week)_] was met, and goserelin 10.8 mg LA every 3 months was deemed non-inferior to goserelin 3.6 mg every month (Table 2) [55].

In a phase III study conducted in Asia, Noguchi et al. compared the efficacy and safety of goserelin 10.8 mg LA every 3 months versus goserelin 3.6 mg every month in premenopausal women with ER-positive advanced breast cancer (Table 1) [51]. The primary endpoint of progression-free survival (PFS) rate at 24 weeks was met, and goserelin 10.8 mg LA every 3 months was deemed non-inferior to goserelin 3.6 mg every month (Table 2) [51].

A phase III study conducted internationally (Study D8664C00008) compared the efficacy, safety, and tolerability of goserelin 10.8 mg LA every 12 weeks versus goserelin 3.6 mg every 4 weeks in premenopausal women with ER-positive advanced breast cancer (Table 1) [61,66]. Only 98 of the planned 260 patients were randomized, so the study had insufficient power to demonstrate non-inferiority of the primary efficacy endpoint: PFS at Week 24 (Table 2) [61].

In a retrospective study conducted 2005–2015, researchers compared the survival outcomes of administering goserelin 10.8 mg LA every 3 months for 2–3 years combined with tamoxifen for 5 years (GnRH-TAM) versus a regimen of adriamycin and cyclophosphamide for 4 cycles followed by tamoxifen for 5 years (AC-TAM) in premenopausal patients with early breast cancer who were HR-positive (Table 1) [63]. The study revealed that both treatment groups exhibited similar DFS outcomes with the 5-year DFS, suggesting that the goserelin 10.8 mg LA every 3 months in GnRH-TAM did not show inferior survival compared to AC-TAM (Table 2) [63].

A retrospective real-world study was conducted comparing goserelin 10.8 mg LA every 3 months to goserelin 3.6 mg monthly administered in 2015–2022 to pre- and perimenopausal HR-positive breast cancer patients (Table 1) [50]. The study assessed E_2_ suppression rates as the primary endpoint, which met the predefined non-inferiority margin (Table 2) [50].

In a retrospective study conducted during the coronavirus disease 2019 (COVID-19) pandemic (2020–2021), the safety and efficacy of administering goserelin 10.8 mg LA every 12 weeks were compared to goserelin 3.6 mg monthly in premenopausal HR-positive breast cancer patients (Table 1) [62]. Overall results revealed similar efficacy outcomes, with both goserelin regimens showing comparable DFS for non-metastatic patients and PFS for metastatic patients (Table 2) [62].

A retrospective 10-year study (2013–2023) of 88 patients who received goserelin with an AI evaluated the OFS failure rate based on having at least one E_2_ level >2.72 pg/mL (Table 1 and Table 2) [64]. Better efficacy, defined as the ability to maintain E_2_ levels ≤ 2.72 pg/mL, was achieved by patients receiving goserelin 10.8 mg LA every 3 months with an AI (52/61, 85.2%) than those receiving goserelin 3.6 mg monthly with an AI (16/27, 59.3%) [64].

In an 8-year real-world study consisting of a retrospective (2015–2021) review of medical chart data and prospective (2022–2023) enrollment of patients from 15 medical centers, goserelin 10.8 mg LA every 3 months was compared to goserelin 3.6 mg monthly in premenopausal patients with HR-positive breast cancer (Table 1) [65]. Non-inferiority of goserelin 10.8 mg LA, every 3 months to 3.6 mg monthly, was established based on analysis of the primary endpoint, the proportion of patients with E_2_ suppression to postmenopausal level at Week 12 ± 4 (Table 2) [65].

## 3. Use Cases for GnRH Agonist LA Formulations in Breast Cancer

The results of the previous studies indicate that both goserelin doses effectively lower estrogen levels in premenopausal women diagnosed with early-stage breast cancer. Goserelin 10.8 mg LA administered every 3 months shows similar effectiveness and safety to goserelin 3.6 mg administered monthly. This interchangeability between the two doses offers flexibility in treatment options, allowing clinicians to choose the most suitable regimen for individual patients. In Table 3, various real-world use cases are described.

## 4. Benefits of the Goserelin 10.8 mg LA Formulation

### 4.1. Goserelin as the GnRH Agonist of Choice for OFS

The selection of GnRH agonists used for OFS includes triptorelin, leuprolide, and goserelin, with choice depending on factors such as patient tolerance and treatment goals [54,67,68]. Although the SOFT and TEXT studies referenced above-utilized triptorelin for pharmacotherapy-induced OFS, goserelin has emerged as one of the preferred choices in modern clinical practice [69]. Historically, the frequent shortages of triptorelin prompted the Canadian Cancer Trials Group (formerly the National Cancer Institute of Canada) to supply goserelin 3.6 mg as an alternative [70]. Clinical experience with goserelin has expanded among oncologists who use it routinely. The oncology nurses appreciate the ease of use with goserelin in ready-to-inject syringes, whereas both leuprolide and triptorelin need to be reconstituted and administered with specialized syringes, which may be prone to occasional malfunction [54,67,68]. This preference for goserelin can also be attributed to its convenience of administration through SC injection rather than intramuscular injections with leuprolide and triptorelin [54,67,68]. Otherwise, efficacy, side effects, and costs are generally regarded as similar between the GnRH agonists [54,67,68]. A meta-analysis of four randomized controlled trials, including SOFT and TEXT studies, did not identify any differences in OFS efficacy between goserelin and triptorelin [71].

### 4.2. Off-Label Use of Goserelin 10.8 mg LA during the Pandemic

Amidst the challenges of the COVID-19 pandemic, there was a notable shift towards the off-label use of goserelin 10.8 mg LA every 3 months among breast cancer patients. Reports indicated that up to 15% of patients transitioned to this treatment modality, a reflection of the COVID-19 response mitigation strategy at British Columbia Cancer Center’s breast tumor group to transition eligible patients to the goserelin 10.8 mg LA formulation [72,73,74]. This action minimized unnecessary visits and facilitated an appropriate response to the increase in neoadjuvant case referrals, enabling the reallocation of resources as needed.

### 4.3. Expanded Indications for Goserelin 10.8 mg LA

Goserelin 10.8 mg LA was recently approved by Health Canada for the expanded indication of managing ER-positive breast cancer with a high risk of recurrence or advanced breast cancer in pre- and perimenopausal women. The evidence summarized above provides data supporting the interchangeability between goserelin 10.8 mg LA every 3 months and 3.6 mg monthly in premenopausal breast cancer patients. A shift in practice is anticipated as access to goserelin 10.8 mg LA standardizes across Canada, with better treatment adherence and a potential impact on outcomes.

### 4.4. More Efficient Healthcare Resource Utilization with Goserelin 10.8 mg LA

Goserelin offers benefits for OFS in breast cancer patients. Utilization of the goserelin 3.6 mg formulation requires monthly injections, which can burden the healthcare system and affect the efficiency of healthcare delivery. Currently, all GnRH agonist injections for breast cancer patients are administered at cancer centers in Canada, consuming significant clerical, nursing, and pharmacy resources and space. Unlike prostate cancer patients who benefit from in-home injection programs with goserelin 10.8 mg LA every 3 months, this resource has until recently been unavailable for breast cancer patients due to off-label use and the impracticality of supporting a monthly home injection program.

The difference in visit frequency between the goserelin 10.8 mg LA and 3.6 mg formulations presents a notable disparity in breast cancer patients’ access to care. Goserelin 10.8 mg LA every 3 months requires 20 cancer center visits, while the 3.6 mg monthly dose requires 60 cancer center visits over the same 5-year adjuvant treatment period. This represents a threefold increase, directly impacting patients’ lives, including additional time off work, escalated transportation expenses, childcare costs, and overall time lost.

Transitioning away from monthly injections as part of a shared decision-making process between patients and their physicians could alleviate these constraints, enabling resources to address the pressing needs of provincial cancer programs, which face shortages in personnel, space, and capacity to support new treatments. Practical considerations, like the discomfort experienced at the injection site due to needle size, underscore the importance of reassessing current practices. Appreciating the patient’s perspective, including the challenges they encounter during treatment, is crucial for enhancing healthcare delivery.

Patients on monthly goserelin who lack access to the long-acting formulation may consider undergoing definitive BSO as an alternative [75]. While this procedure achieves permanent ovarian function suppression, it comes with long-term risks [75,76]. Premature ovarian function suppression is associated with increased risks of cardiovascular disease, increased risk of osteoporosis, cognitive decline, and higher all-cause mortality [75,76]. Furthermore, surgery itself also carries inherent risks, including complications related to anesthesia, bleeding, infection, and potential damage to surrounding structures [75,77].

## 5. Recommendations for Monitoring Patients on GnRH Agonist LA Formulations in Breast Cancer

The efficacy of GnRH agonist LA formulations should be evaluated based on menstrual function, with monitoring of E_2_, LH, and FSH hormone levels to ensure appropriate dosing. Adherence is based on nursing assessment prior to each injection, every 3 months. There are no absolute contraindications. Data regarding the use of OFS plus AIs (which stimulate ovarian function) in very young women, particularly those who did not receive OFS-inducing chemotherapy, is somewhat limited; monitoring the menopausal profiles of these patients may be of value at the treating physician’s discretion.

## 6. Future Directions

Ongoing studies continue to include the goserelin 10.8 mg LA every 3 months formulation among the standard of care options for OFS [26]. In the phase III OFSET (NRG-BR009) trial, 3960 patients will be randomized over eight years to receive either OFS (a GnRH agonist) + AI for five years or undergo adjuvant chemotherapy followed by OFS + AI [9]. The selection of GnRH agonists is at the investigator’s discretion and may include either the monthly or LA formulation administered every 3 months of goserelin, leuprolide, or triptorelin [26]. The primary endpoint of the trial is the improvement of invasive breast cancer-free survival, with secondary endpoints including various measures of survival and evaluation of menopausal symptoms [9]. Results will help determine whether OFS will abrogate the need for chemotherapy in premenopausal women with HR-positive, node-positive breast cancer.

Another ongoing study evaluating long-acting GnRH agonists is the Ovarian Suppression Evaluating Subcutaneous Leuprolide Acetate in Breast Cancer (OVELIA) trial [78]. This phase III single-arm, open-label, 48-week study will evaluate a 3-month injectable suspension as the OFS + tamoxifen or an AI in patients with HR-positive, HER2-negative breast cancer [78]. Enrollment is limited to 18–49-year-old patients (men and women) with a body mass index of 18.0–35.0 kg/m^2^ [78].

## 7. Conclusions

Effectively managing breast cancer in premenopausal women requires a comprehensive approach, considering patient preferences, treatment efficacy, and sustainability within healthcare systems. Preserving ovarian function is crucial, with goserelin emerging as the preferred option for OFS due to its convenience and established safety profile. Goserelin is widely used in clinical trials involving premenopausal and peri-menopausal women, with interchangeable doses offering flexibility in treatment selection.

Goserelin 10.8 mg LA provides additional convenience for patients while potentially enhancing satisfaction and adherence to therapy. Patient demand underscores the need for access to this formulation. While provinces like British Columbia, Ontario, and Quebec offer off-label access to goserelin 10.8 mg LA, availability varies due to provincial restrictions, emphasizing the need for approval and provincial funding to standardize and ensure equal treatment access throughout Canada.

With the recent approval of goserelin 10.8 mg LA by Health Canada on 6 May 2024 for use in breast cancer, Canada is the latest to join over 60 countries worldwide to expand the accepted indications for goserelin LA and ensure its availability to potentially enhance healthcare delivery, patient care, and breast cancer outcomes. This change ensures consistent access to this therapy nationwide and recognizes its efficacy and safety for treating breast cancer in premenopausal females.

## Figures and Tables

**Table 1:** Overview of studies supporting the use of goserelin 10.8 mg LA every 3 months in breast cancer.

Study	Study Design	Treatment Arms	Patient Population	Endpoints
Masuda et al., 2011 [55] Phase II (D8664C00004/NCT 00303524)	Open-label, randomized, parallel group, multicenter	Goserelin 10.8 mg LA Q3 months or Goserelin 3.6 mg Q month + Tamoxifen 20 mg PO QD	Premenopausal women * with ER+ early breast cancer who had undergone radical surgery *n* = 170 (1:1 randomization)	Primary (non-inferiority): AUC_(4–24 week)_ of E_2_ serum concentration
				Key secondary: E_2_ serum concentrations; percentage of patients with mean E_2_ serum concentration ≤30 pg/mL; menstruation; safety
Noguchi et al., 2016 [51] Phase III (D8666C00001/NCT 01073865)	Open-label, randomized, parallel group, multicenter	Goserelin 10.8 mg LA Q3 months or Goserelin 3.6 mg Q month + Tamoxifen 20 mg PO QD	Premenopausal women ^†^ with ER+ advanced breast cancer *n* = 222 (1:1 randomization)	Primary (non-inferiority): PFS at Week 24
				Key secondary: ORR at Week 24; E_2_ serum concentrations; safety
Collaborative Data on file [61] Phase III (D8664C00008)	Open-label, randomized, parallel group, multicenter	Goserelin 10.8 mg LA Q3 months or Goserelin 3.6 mg Q month + Tamoxifen 20 mg PO QD	Premenopausal women ^‡^ with ER+ advanced breast cancer *n* = 98 (1:1 randomization)	Primary (non-inferiority): PFS at Week 24
				Key secondary: ORR at Week 24; E_2_ serum concentrations
Sa-Nguanraksa et al., 2019 [63]	Retrospective, single center	Goserelin 10.8 mg LA Q3 months + Tamoxifen 20 mg PO QD or AC-TAM	Premenopausal women ^§^ with HR+ breast cancer *n* = 170	DFS
Wu et al., 2023 [50]	Retrospective, observational, real-world electronic record review	Goserelin 10.8 mg LA Q3 months or Goserelin 3.6 mg Q month	Pre- and perimenopausal patients with HR+ breast cancer *n* = 240	Primary (non-inferiority): E_2_ serum concentrations
				Key secondary: OS, DFS for early breast cancer, PFS for advanced breast cancer
El Zawawy et al., 2022 [62]	Retrospective review	Goserelin 10.8 mg LA Q12 weeks or Goserelin 3.6 mg Q month + Tamoxifen 20 mg PO QD or an AI **	Premenopausal women with HR+ breast cancer *n* = 87	E_2_ serum concentrations; DFS for non-metastatic breast cancer; PFS for metastatic breast cancer; safety
Blotta et al., 2023 [64]	Retrospective	Goserelin 10.8 mg LA Q3 months or Goserelin 3.6 mg Q month + AI	Premenopausal women with ER+ breast cancer *n* = 88	Patients with E_2_ level >2.72 pg/mL (ineffective OFS)
Wang et al., 2024 [65] (NCT 05184257)	Retrospective/prospective, real-world, multicenter, medical record data review	Goserelin 10.8 mg LA Q3 months or Goserelin 3.6 mg Q month ± SERM/AI	Premenopausal women with HR+ breast cancer *n* = 590	Primary (non-inferiority): E_2_ suppression to postmenopausal level at Week 12 ± 4

AC-TAM, adriamycin + cyclophosphamide followed by tamoxifen; AI, aromatase inhibitor; AUC_(4–24 week)_, area under the serum concentration-time curve from week 4 to week 24; DFS, disease-free survival; E_2_, estradiol; ER+, estrogen receptor-positive; FSH, follicle stimulating hormone; HR+, hormone receptor-positive; LA, long-acting; OFS, ovarian function suppression; ORR, objective response rate; OS, overall survival; PFS, progression-free survival; PO, per oral; Q month, every month; Q3 months, every 3 months; QD, daily; SERM, selective estrogen receptor modulator. * Premenopausal defined as menses within one year, and E_2_ ≥ 10 pg/mL, and FSH ≤ 30 mIU/mL within three weeks prior to randomization. ^†^ Premenopausal is defined as (1) menses within one year and (2) E_2_ ≥ 10 pg/mL and FSH ≤ 30 mIU/mL within four weeks prior to randomization. For patients who had a hysterectomy, it was acceptable to meet only criterion 2. ^‡^ Premenopausal defined as (1) menses within one year of administration of study drug, and (2) E_2_ ≥ 10 pg/mL and FSH ≤ 30 mIU/mL within four weeks of administration of study drug. For patients who had a hysterectomy, it was acceptable to meet only criterion 2. ^§^ Premenopausal defined as menses within one year before surgery or had FSH < 30 IU/mL. ** Tamoxifen was received by 43% of patients, and AI was received by 57% of patients.

**Table 2:** Endpoints of studies supporting the use of goserelin 10.8 mg LA every 3 months vs. 3.6 mg every month in breast cancer.

Study	E_2_ Levels	PFS	ORR	DFS	Other Endpoints	Safety (10.8 mg vs. 3.6 mg)
Masuda et al., 2011 [55]	Primary endpoint (non-inferiority) 10.8 mg: AUC_(4–24 week)_ 18.32 pg/mL•week 3.6 mg: AUC_(4–24 week)_ 18.95 pg/mL•week Ratio = 0.974 (95% CI: 0.80, 1.19) *	NR	NR	10.8 mg: 4 events in 675 days 3.6 mg: 1 event in 676 days	Menstruation ∘ Week 4—10.8 mg: 67.4%; 3.6: 72.6% ∘ Week 8—10.8 mg: 2.3%; 3.6: 1.2% ∘ Week 12—10.8 mg: 0%; 3.6: NR ∘ Week 16—10.8 mg: 0%; 3.6: 0% FSH levels after 4 weeks: 1.3–1.9 mIU/mL for both groups Goserelin PK for 10.8 mg ∘ Geometric mean C_max_: 4.5 ng/mL ∘ T_max_: 2.4 h	Any AE: 97.6% vs. 97.6% Most common AEs ∘ Hot flush: 69.4% vs. 63.5% ∘ Headache: 16.5% vs. 15.3% ∘ Arthralgia: 14.1% vs. 16.5% ∘ Hyperhidrosis: 11.8% vs. 17.6%
	≥98.8% in both groups maintained E_2_ < 30 pg/mL *(postmenopausal range)*					
Noguchi et al., 2016 [51]	Week 12 10.8 mg: 26.3 pg/mL 3.6 mg: 25.4 pg/mL	Primary endpoint at Week 24 (non-inferiority) ^†^ 10.8 mg: 61.5% 3.6 mg: 60.2% Treatment difference 1.3 (95% CI: –11.4, 13.9)	Week 24 10.8 mg: 23.9% 3.6 mg: 26.9% Treatment difference 3.0% (95% CI: –15.5%, 9.7%)	NR	NR	Any AE: 65.7% vs. 63.7% Most common AEs ∘ Hot flush: 13.9% vs. 19.5% ∘ Nasopharyngitis: 12.0% vs. 8.0% ∘ Headache: 6.5% vs. 6.2% ∘ Back pain: 4.6% vs. 8.0%
	Week 24 10.8 mg: 20.3 pg/mL 3.6 mg: 24.8 pg/mL					
Collaborative Data on file [61]	10.8 mg: 7.2 pmol/L 3.6 mg: 10.4 pmol/L	Primary endpoint at Week 24 (non-inferiority) ^‡^ 10.8 mg: 69.4% 3.6 mg: 73.5% Treatment difference –4.1 (95% CI: –21.4, 13.6)	Week 24 10.8 mg: 28.9% 3.6 mg: 25.6%	NR	NR	Any AE: 78.0% vs. 77.1% Most common AE ∘ Hot flush 38.0% vs. 37.5%
Sa-Nguanraksa et al., 2019 [63]	NR	NR	NR	5-year DFS GnRH-TAM: 0.97 AC-TAM: 0.98	NR	NR
				10-year DFS GnRH-TAM: 0.97 AC-TAM: 0.94 *p* = 0.86 ^§^ hazard ratio = 1.90 (95% CI: 0.19, 18.80)		
Wu et al., 2023 [50]	Primary endpoint (non-inferiority) E_2_ suppression rate: 10.8 mg: 99.0% 3.6 mg: 92.7% Risk difference = 0.065 (95% CI: 0.021, 0.135) ** p = 0.0187	3.2-year PFS 10.8 mg: 77.1% 3.6 mg: 80.0%	NR	5-year DFS 10.8 mg: 99.0% 3.6 mg: 96.7%	5-year OS—10.8 mg: 96.6%; 3.6: 93.8%	NR
El Zawawy et al., 2022 [62]	Week 12 10.8 mg: 15.4 pg/mL 3.6 mg: NR	Metastatic 10.8 mg: 66.7% 3.6 mg: 63.6% *p* = 0.88 NS	NR	Non-metastatic 10.8 mg: 86.2% 3.6 mg: 87.1% *p* = 0.71 NS	Amenorrhea—10.8 mg: 100%; 3.6: 100% OS—10.8 mg: 100%; 3.6: 100%	Most common AEs ∘ Hot flush: 65.8% vs. 66.7% ∘ Headache: 36.6% vs. 40.5% ∘ Arthralgia: 26.8% vs. 28.5% ∘ Hyperhidrosis: 7.3% vs. 7.1%
	Week 24 10.8 mg: 10.8 pg/mL 3.6 mg: NR					
	Week 36 10.8 mg: 9.6 pg/mL 3.6 mg: NR					
Blotta et al., 2023 [64]	E_2_ level > 2.72 pg/mL (ineffective OFS): 10.8 mg: 14.8% 3.6 mg: 40.7% *p* = 0.007	NR	NR	NR	NR	NR
Wang et al., 2024 [65]	Primary endpoint (non-inferiority) E_2_ suppression to postmenopausal level at Week 12 ± 4 10.8 mg: 99.1% 3.6 mg: 95.3% Difference = 3.8 (95% CI: 0.6, 8.1) ^††^	NR	NR	NR	NR	NR

AC-TAM, adriamycin + cyclophosphamide followed by tamoxifen; AUC_(4–24 weeks)_, area under the serum concentration-time curve from week 4 to week 24; AE, adverse event; CI, confidence interval; C_max_, maximal concentration; DFS, disease-free survival; E_2_, estradiol; FSH, follicle stimulating hormone; GnRH-TAM, gonadotropin-releasing hormone agonist plus tamoxifen; LA, long-acting; NR, not reported; NS, not significant; OFS, ovarian function suppression; ORR, objective response rate; OS, overall survival; PFS, progression-free survival; PK, pharmacokinetics; T_max_, time at maximal concentration. * Non-inferiority criteria were met; they were below the upper limit of 1.118. ^†^ Non-inferiority criteria met; lower 95% CI was above the pre-defined margin of –17.5%. ^‡^ Non-inferiority criteria not met; recruitment was terminated prematurely. Therefore, the study was no longer adequately powered to detect non-inferiority. ^§^ Log rank test; *p* = 0.58 based on Cox regression. ** Non-inferiority criteria were met within a margin of –15%. ^††^ Non-inferiority criteria met; lower 95% CI was above the pre-defined margin of –10%.

**Table 3:** Use cases for goserelin 10.8 mg LA administered every 3 months.

**A**	**Improving patient quality of life with transition from goserelin 3.6 mg monthly to 10.8 mg LA every 3 months**
A 39-year old premenopausal woman with a post-partum tumor stage pT2 (28 mm), lymph node stage N1a (2/5 nodes involved), no evidence of distant metastases (M0) grade 2 ER (>90%)/PR (80%)-positive, HER2-negative, Ki-67 18%, extensive LVI and PNI positive, invasive ductal carcinoma of the breast. She was treated with adjuvant ddACT chemotherapy, OFS with goserelin 3.6 mg SC monthly, AI therapy with letrozole, zoledronic acid and adjuvant radiotherapy. Subsequently began adjuvant CDK4/6 inhibitor therapy with ribociclib. After 3 months on ribociclib, she returned to work on a progressive schedule. She found it challenging to go back to being a full time working mom and coming in for monthly appointments. After 1 year of goserelin 3.6 mg SC with established ovarian function suppression biochemically based on FSH and E_2_, she was transitioned to 10.8 mg SC every 12 weeks. She feels much happier with this schedule and reports an improvement in quality of life as a result, with less stress and fewer visits to the cancer center. Consideration can be made for the home injection program for further alleviation of burden on the patient.	
**B**	**Initiating OFS with goserelin 10.8 mg LA every 3 months**
A 47-year-old premenopausal woman who presented with a self-detected left-sided clinical tumor stage T3, lymph node negative (N0), grade 1, ER (>90%)/PR (>90%)-positive, HER2-negative, Ki-67 low 5%, invasive lobular carcinoma of the breast. It measured 5.1 cm in maximal dimension on clinical examination, although the breast magnetic resonance imaging suggested an extent of over 7.1 cm. There were additional microcalcifications in the right breast. She elected to have bilateral mastectomies with immediate reconstruction. The final pathology revealed pT3N2a, grade 2 (mitotic rate 1), 39 mm, 19 mm and 12 mm foci of invasive lobular carcinoma which were adjacent and represented an aggregate of 7 cm of disease with 4/12 nodes involved (10 mm deposit, no ENE). Final surgical margins were positive in the anterior and superior, with LVI present. Staging computed tomography of the chest, abdomen, pelvis revealed no abnormalities. The patient completed adjuvant chemotherapy with the ddACT protocol. Locoregional radiotherapy was completed in March 2023. She started OFS with goserelin 10.8 mg SC, and 4 weeks later added AI therapy with letrozole and then adjuvant CDK4/6 inhibitor therapy with abemaciclib. She had regular monthly menstrual cycles and no symptoms to suggest perimenopausal state prior to presentation. While it is likely that she had chemotherapy-induced ovarian failure, it was decided to offer OFS but at the 10.8 mg dose upfront with iterative approach to add on nonsteroidal AI. Tamoxifen should be avoided with abemaciclib given the higher risk of venous thromboemlism and thus this was the best choice for the patient who also lives in a remote area with a long commute into town.	

AI, aromatase inhibitor; CDK4/6, cyclin-dependent kinase 4 and 6; ddACT, dose-dense adriamycin/cyclophosphamide/paclitaxel; E_2_, estradiol; ENE, extranodal extension; ER, estrogen receptor; FSH, follicle stimulating hormone; HER2, human epidermal growth factor receptor 2; Ki-67, antigen Kiel 67; LA, long-acting; LVI, lymphovascular invasion; OFS, ovarian function suppression; PNI, perineural invasion; PR, progesterone receptor; SC, subcutaneous.

## Data Availability

No new data were created or analyzed in this study. Data sharing is not applicable to this article.

## Supplementary Materials

The following supporting information can be downloaded at: https://www.mdpi.com/article/10.3390/curroncol31080314/s1, Table S1: Overview of studies supporting the use of other GnRH agonists every 3–6 months in breast cancer. Table S2: Endpoints of studies supporting the use of other GnRH agonists every 3–6 months in breast cancer.
