# Supplementary material for: An Overview of Long-Acting GnRH Agonists in Premenopausal Breast Cancer Patients: Survivorship Challenges and Management
Source: Curr Oncol. 2024 Jul 25;31(8):4209–24. doi: 10.3390/curroncol31080314 (PMC11352532; doi:10.3390/curroncol31080314)
Supplement: Supplementary file 1 [file curroncol-31-00314-s001.zip › curroncol-3062301-supplementary.pdf]

Table S1. Overview of studies supporting use of other GnRH agonists every 3–6 months in breast cancer.

Study	Study Design	Treatment Arms	Patient Population	Endpoints
TABLE Study<br>Schmid et al 2007<br>Schmid et al 2002	Phase III, open-label, randomized, multicenter	Leuprolide 11.25 mg Q3 months x 2 years<br>or CMF x 6 courses	Premenopausal patients with ER+ breast cancer*<br>$n = 589$	Primary: RFS<br>Secondary: OS, AEs, E_2 suppression, menstrual status
Shiba et al 2016<br>Kurebayashi et al 2021	Open-label, randomized, multicenter	Leuprolide 11.25 mg Q3 months x 2 years or x 3–5 years<br>+<br>Tamoxifen 20 mg PO QD	Premenopausal patients ≥ 20 years old with ER+ or PR+ breast cancer<br>$n = 222$	Primary: DFS and 2-year landmark DFS<br>Secondary: OS, 2-year landmark OS, menstruation status, quality of life, E_2/LH/FSH levels, safety
Takeda<br>(NCT02154139)	Observational, prospective, cohort, multicenter	Leuprolide 11.25 mg Q12 weeks	Premenopausal women with advanced or recurrent breast cancer who received adjuvant therapy<br>$n = 644$	Primary: Number of patients reporting ≥ 1 AE; number of patients reporting ≥ 1 SAE<br>Secondary: Percentage of patients with best response (CR or PR), PFS, RFS
Kurebayashi et al 2017	Phase III, open-label, randomized, parallel-group	Leuprolide 22.5 mg Q24 weeks<br>or Leuprolide 11.25 mg Q12 weeks<br>+<br>Tamoxifen 20 mg PO QD	Premenopausal women ≥ 20 years old with ER+ or PR+ breast cancer<br>$n = 150$	Primary: E_2 suppression rate (≤ 30 pg/mL) Week 4–48<br>Secondary: E_2/LH/FSH levels, DFS, distant DFS, pharmacokinetic data, safety
Kendzierski et al 2018	Retrospective, single-center	Leuprolide 22.5 mg Q3 months<br>or Leuprolide 7.5 mg Qmonth<br>+<br>Aromatase inhibitor	Premenopausal patients ≥ 18 years old with ER+ or PR+ breast cancer†<br>$n = 201$	Primary: Proportion of patients achieving ovarian ablation (E_2 < 40 pg/mL and FSH 23-116 mU/mL) after 3 months<br>Secondary: DFS at 1 year, OS at 1 year, AEs
Lee et al 2020	Retrospective chart review	Leuprolide 11.25 mg Q3 months<br>+<br>Tamoxifen	Premenopausal women with HR+ breast cancer<br>$n = 318$	E_2 level, menstrual status, drug discontinuation due to AEs, AEs
Wu et al 2021	Retrospective chart review	Leuprolide 22.5 mg Q6 months<br>+<br>Tamoxifen	Premenopausal women with HR+ breast cancer<br>$n = 228$	E_2 level, menstrual status, drug discontinuation due to AEs, AEs
Chen et al 2024	Retrospective, single-center, real-world study	Leuprolide 11.25 mg Q3 months<br>or Leuprolide 3.75 mg Qmonth<br>or Goserelin 3.6 mg Qmonth<br>+<br>Aromatase inhibitor	Premenopausal women with HR+ breast cancer<br>$n = 264$	Primary: E_2 ≥ 30 pg/mL (insufficient OFS)<br>Secondary: Effectiveness & risk factors for insufficient OFS

AE, adverse event; CMF, cyclophosphamide/methotrexate/fluorouracil; CR, complete response; DFS, disease-free survival; E_2, estradiol; ER+, estrogen receptor-positive; FSH, follicle-stimulating hormone; GnRH, gonadotropin-releasing hormone; HR+, hormone receptor-positive; LH, luteinizing hormone; OFS, ovarian function suppression; OS, overall survival; PFS, progression-free survival; PO, orally; PR, partial response; PR+, progesterone receptor-positive; Q3 months, every three months; Q6 months, every six months; Q12 weeks, every 12 weeks; Q24 weeks, every 24 weeks; QD, daily; Qmonth, every month; RFS, recurrence-free survival; SAE, serious adverse event; TABLE, Takeda Adjuvant Breast cancer study with Leuprorelin acetatE.

*Initially, patients with unknown ER status tumors were allowed until an amendment was issued in March 1998 to require ER+

†Status post either (1) total mastectomy ± radiotherapy or (2) breast-conserving surgery with radiotherapy, and on adjuvant ovarian suppression + aromatase inhibitor therapy x ≥1 year

Table S2. Endpoints of studies supporting use of other GnRH agonists every 3–6 months in breast cancer.

Study	E_2 Levels	DFS / PFS	Other Endpoints	Safety
TABLE Study<br>Schmid et al 2007<br>Schmid et al 2002	1-year $E_2 \leq 30$ pg/mL:<br>11.25 mg: 92.1% vs CMF: 66.2%	NR	• 5-year RFS: 11.25 mg: 63.9% vs CMF: 63.4%<br>• 5-year OS: 11.25 mg: 81.0% vs CMF: 71.9%<br>• 1-year amenorrhea: 11.25 mg: 88.0% vs CMF: 43.9%	• AEs: 11.25 mg: hot flashes, increased sweating vs CMF: nausea, vomiting, diarrhea, asthenia, alopecia
Shiba et al 2016<br>Kurebayashi et al 2021	E_2 levels < 30 pg/mL within 12 weeks & remained low for both groups	DFS<br>2-year group: 82.0%<br>3–5 year group: 86.6%<br><br>2-year landmark DFS<br>2-year group: 83.6%<br>3–5 year group: 81.6%	• OS – 2-year group: 98.9% vs 3–5 year group: 96.9%<br>• 2-year landmark OS – 2-year group: 97.4% vs 3–5 year group: 98.9%	• Bone-related AEs* – 2-year group: 11.6% vs 3–5 year group: 16.4%<br>• Most common AEs<br>○ Hot flush: 2-year group: 58.9% vs 3–5 year group: 59.1%<br>○ Hyperhydrosis: 2-year group: 25.0% vs 3–5 year group: 27.3%<br>○ Arthralgia: 2-year group: 9.8% vs 3–5 year group: 21.8%<br>○ Headache: 2-year group: 10.7% vs 3–5 year group: 20.9%<br>○ Injection site induration: 2-year group: 17.0% vs 3–5 year group: 18.2%
Takeda<br>(NCT02154139)	NR	PFS at week 96: 49.7%	• Best response at week 96: 15.0%<br>• RFS at week 96: 95.4%	• AEs: 128<br>• Most common AEs<br>○ Injection site induration: 6.4%<br>○ Hot flush: 5.4%<br>• SAEs: 3.1%
Kurebayashi et al 2017	Primary endpoint (non-inferiority)<br>E_2 suppression rate (≤ 30 pg/mL) Week 4–48:<br>22.5 mg: 97.6%<br>11.25 mg: 96.4%<br>Difference = 1.2%<br>(95% CI: –5.2, 7.8)†	DFS at week 96:<br>22.5 mg: 97.3%;<br>11.25 mg: 97.5%<br><br>Distant DFS at week 96:<br>22.5 mg: 98.5%;<br>11.25 mg: 98.8%	• Median E_2 suppressed to 0 pg/mL Week 4–48, remained low through Week 96<br>• Median LH suppressed to ≤ 1 mIU/mL Week 4–96<br>• Median FSH suppressed to ≤ 2.5 mIU/mL Week 4–96	• Most common AEs (22.5 mg vs 11.25 mg)<br>○ Nasopharyngitis: 56.6% vs 50.0%<br>○ Hot flush: 51.8% vs 57.1%<br>○ Injection site induration: 43.4% vs 39.3%<br>○ Radiation skin injury: 37.3% vs 46.4%
Kendzierski et al 2018	NR	DFS at 1 year:<br>22.5 mg: 96% vs<br>7.5 mg: 95%<br>p = 0.75	• Primary endpoint – Ovarian ablation: 22.5 mg: 99% vs 7.5 mg: 100%, p = 1.00<br>• OS: 22.5 mg: 99% vs 7.5 mg: 100%, p = 1.00	• Most common AEs (22.5 mg vs 7.5 mg)<br>○ Musculo-skeletal pain: 89.1% vs 92%<br>○ Hot flush: 86.1% vs 83%<br>○ Fatigue: 65.3% vs 68.0%<br>○ Insomnia: 52.5% vs 56.0%
Lee et al 2020	Mean E_2 level: 4.9 pg/mL	NR	• Amenorrhea: 100%<br>○ Vaginal bleeding in 4 patients determined to not be of menstrual origin	• No discontinuation or change in treatment due to SAEs
Wu et al 2021	Mean E_2 level: 5.5 pg/mL<br>E_2 < 30 pg/mL: 99.6%	NR	• Amenorrhea: 100%<br>○ Vaginal bleeding in 12 patients (5.3%) determined to not be of menstrual origin	• No discontinuation or change in treatment due to SAEs

Chen et al 2024	$E_2 \geq 30$ pg/mL:<br>11.25 mg: 7.14%<br>3.75 mg: 6.35%<br>3.6 mg: 7.69%<br>p = 0.900	NR	• Risk factor for insufficient OFS: age ≤ 40 years (OR = 0.900, 95% CI: 0.824, 0.982, p = 0.018)	NR

AE, adverse event; CMF, cyclophosphamide/methotrexate/fluorouracil; DFS, disease-free survival; E_2, estradiol; FSH, follicle-stimulating hormone; GnRH, gonadotropin-releasing hormone; LH, luteinizing hormone; NR, not reported; OFS, ovarian function suppression; OS, overall survival; PFS, progression-free survival; RFS, recurrence-free survival; SAE, serious adverse event; TABLE, Takeda Adjuvant Breast cancer study with Leuprorelin acetatE.

*Osteoporosis, osteopenia, or bone fracture.

†Non-inferiority criteria met; lower 95% CI was above the pre-defined margin of –10%.
